# Students’ perspectives of factors related to delayed completion of online RN-BSN programs

**DOI:** 10.1186/s12912-021-00574-7

**Published:** 2021-04-07

**Authors:** Kechinyere C. Iheduru-Anderson

**Affiliations:** School of Rehabilitation and Medical Sciences, the Herbert H. and Grace A. Dow College of Health Professions, Central Michigan University, 1280 E Campus Dr, Mount Pleasant, MI 48859 USA

**Keywords:** Nursing education, Advancement, Employment, Barriers, Registered nurse, Bachelor of science, Online education

## Abstract

**Background:**

There are multiple educational programs for individuals to become registered nurses (RNs), and the transition from an associate degree or diploma to a Bachelor of Science in Nursing (BSN) degree is vital to healthcare. This study examined the factors contributing to delayed completion or withdrawal from online RN-BSN nursing programs from students’ perspectives.

**Method:**

Thematic content analyses were conducted on qualitative data obtained from semi-structured interviews (*N* = 26).

**Results:**

Complex factors contributed to delayed completion of online RN-BSN programs, including student-, institutional-, and faculty-related factors.

**Conclusion:**

This study validated and extends previous studies without delineating students in fully online programs from hybrid and face-to-face programs. Students, faculty, and institutions all have a role to play in facilitating on-time program completion. Recognizing and mitigating the barriers that delay on-time program completion is required to foster nurses to obtain a BSN degree.

**Supplementary Information:**

The online version contains supplementary material available at 10.1186/s12912-021-00574-7.

## Background

Unlike many other health professional programs with standardized educational requirements for entry to practice, nursing has multiple educational programs for registered nurses (RNs): hospital or diploma school programs, a two-year junior college or associate degree (AD) program, and a four-year baccalaureate or university program leading to a Bachelor of Science in Nursing (BSN) degree. In a December 1965 position statement, the American Nurses Association (ANA) declared that education for professional nurses should take place in institutions of learning within the education general system of. The ANA then recommended that a baccalaureate degree become the entry educational requirement for nursing practice, while AD education in nursing be recognized as the beginning of technical nursing practice [12]. This recommendation ignited debates about entry into nursing practice that are still ongoing.

Decades later, the American Association of Colleges of Nursing (AACN) [7] recommended that baccalaureate-level education in nursing become the minimum educational requirement for professional nursing practice. This was followed by the National Academy of Medicine formally Institute of Medicine’s (IOM) recommendation to increase the proportion of RNs with a baccalaureate degree to 80% by 2020, [34]. RNs’ transition from an AD or diploma to a BSN is vital to healthcare organizations because studies indicate there is a link between BSN-prepared RNs and improved patient outcomes [2, 3, 13], higher job satisfaction [46], critical thinking, and leadership skills [19, 50]. “The additional course work [in the BSN program] enhances the student’s professional development; prepares the new nurse for a broader scope of practice; and provides the nurse with a better understanding of the cultural, political, economic, and social issues that affect patients and influence healthcare delivery” [8], p. 5).

Granting the goal of 80% BSN-prepared nurses by 2020 was not reached, tremendous progress has been made with 57% achieved in 2019. Prior to 1965, hospital-based diploma programs were responsible for the education of approximately 80% of nurses [36]. Although the number of first-time National Council Licensure Examination (NCLEX) test takers with a BSN degree has steadily increased recently, AD remains the most commonly reported route for initial nursing education in the U.S. (National Council of State Boards of Nursing [42]. As reported by the Campaign for Action [16], currently, the percentage of RNs with at least a BSN degree is approximately 57%—an 8% increase from 2010. Associate- and diploma-educated first-time NCLEX test takers accounted for 51.5% [18]. Of the 139,792 BSN graduates from accredited nursing programs in the U.S., 66,369 (47.5%) were RN to BSN (RN–BSN) graduates, compared to 30.6% in 2010 [17].

Despite the ongoing controversy about the entry points into professional nursing, it is agreed that there is a need to increase the number of BSN-prepared nurses in the U.S. This led to the RN–BSN program as a bridge to promote less-educated nurses’ upward career mobility. Many states in the U.S. have adopted legislation to increase the number BSN-prepared nurses; e.g., New York’s “BSN in 10 Law” requires future nurses graduating from an AD or diploma nursing program to obtain BSN qualification within 10 years of their initial RN license [41]. The “BSN in 10 Law” bill expands the education of RNs to the baccalaureate level, while still maintaining multiple entry points into the profession. Online RN–BSN programs has proliferated over the past two decades. According to the AACN [9], there are 777 RN–BSN programs available nationwide, including more than 600 programs that are offered at least partially online. The quality of nursing programs within an academic institution are demonstrated by the achievement of accreditation from professional organizations. Currently three major accrediting bodies for nursing education programs exists: Commission on Collegiate Nursing Education (CCNE), Accreditation Commission for Education in Nursing, and Commission for Nursing Education Accreditation [10]. Schools and programs must define the time frame for program completion [21]. For example, the CCNE [21] requires all programs to list an intended graduation rate, and a completion rate of 70% is required. RN–BSN students enrolled in the program must successfully complete the BSN degree within the delineated timeframe set by the school or program. Failure to meet the rates must be reported to the CCNE with a corrective action report.

There are several formats for online RN–BSN programs: 8-week term/semester, 7-week term/semester, 10-week term/semester, and 15−/16-week term/semester. Online programs are designed for working nurses to control their time, offer students flexibility, and provide 24-h access to course materials. However, there may be challenges present that prolong or hinder degree completion. Not only is the on-time completion rate of RN–BSN programs important for meeting IOM recommendation, it is vital for nursing program’s accreditation. Therefore, it is critical to understand the factors that contribute to delayed completion or failure to complete RN–BSN programs from students’ perspectives.

Significant literature exists regarding the barriers related to returning for completion of RN–BSN education [24, 25, 38, 40, 43, 49]. Lack of incentive in some settings such as the workplace culture and lack of distinction between the functions of BSN- and AD-prepared nurses are barriers to degree advancement [43]. Lack of managerial and employer support, financial and family commitments, and lack of peer support are also contributing factors that hinder return to or completion of RN–BSN programs [6, 14, 25, 43, 44]. The work environment, pressure from employers, and peer pressure also contribute to nurses’ decisions to return to school for their RN–BSN [26]; while employer financial support, managers’ emotional support, and flexible work schedules are cited as supportive [38].

RN–BSN programs are bridge programs that should build upon nurses’ prior education and experience [9] and provide RNs with the education necessary to advance their careers. Many cite lack of credit transfer as a barrier. Although there have been large-scale articulation agreements between university programs and two-year colleges offering associates degree to facilitate seamless transition to BSN education [45, 51], there has been poor recognition for prior academic work [14, 38]. Scant literature has examined the factors contributing to the delays or withdrawal from online RN–BSN programs from students’ perspectives. Many online RN–BSN programs include a program that can be completed in 2 years or less. Consequently, the current study focused on current or previous online RN–BSN students who took more than 2 years to complete their program.

### Study purpose

The purpose of this descriptive qualitative study were a) to elucidate the factors contributing to delayed completion or withdrawal from online RN–BSN programs from students’ perspectives to determine ways to alleviate said barriers; b) to answer two important questions: i) What factors led to the decision to return to school for an online RN–BSN Program? ii) What were the most important factors online RN-BSN students considered before choosing which school to attend?

## Method

### Research design and ethical considerations

This study employed a descriptive qualitative design using semi-structured interviews conducted virtually via telephone and WebEx. Permission to conduct this study was granted by the Central Michigan University Institutional Review Board (IRB). An IRB approved informed consent to participate in an interview form with detailed information about the study was emailed to participants who contacted the researcher for more information. Prior to each interview, the consent form was reviewed with participants who then provided verbal consent to participate as part of the audio recording. Pseudonyms which were used for data collection, analysis, and presentation of the findings were assigned to each participant to ensure confidentiality.

### Sampling

Recruitment began by inviting participants from a convenience sample of individuals known to the author via emails, LinkedIn, and Facebook. The author sent emails to colleagues in different institutions requesting that they post the flyers and forward emails to their students. Snowball sampling was used to identify other potential participants by asking participants to forward recruitment flyers to their peers and students. This recruitment strategy resulted in participants from seven U.S. states.

A four-question screening form was used to determine prospective participants’ eligibility. Any individual meeting the eligibility criteria was contacted by telephone and invited to participate in a 30–45-min interview. Inclusion criteria were being an RN and currently enrolled in or having completed an online RN–BSN program that has taken/took more than 2 years to complete. Students who have not been in an online RN—BSN programs longer than 2 years, are in face-to-face or hybrid programs, or in prelicensure BSN program were excluded from the study. Participation was voluntary, participants can withdraw at any time from the study and were not compensated for their participation.

### Data collection

Participants’ demographic information was collected using a short IRB approved demographic data collection form developed by the researcher for this study, including age, sex, ethnicity, number of years as a nurse, setting of primary practice, and number of years in the RN–BSN program. Twenty-six (aged 29–52 years; 7 men and 19 women) nurses participated. Their experience as an RN ranged 5–23 years (Table 1).

**Table 1:** Participant Demographic Characteristics (*N* = 26)

Characteristics	n
**Sex**	
Male	7
Female	19
Age range in years	29–52
Number of years as a nurse (range)	5 to 23
**Ethnic identification**	
Hispanic/Latino (nonwhite)	3
Black/African American	11
White (non-Hispanic)	10
Two or more race	2
**BSN education status**	
Completed > 2 years	6
Program in progress > 2 years	16
Withdrew from program	4
**Setting of clinical practice**	
LTACH - Long term Acute Care Hospital	2
LTCF - Long Term Care Facility	5
ACH - Acute Care Hospital	17
HCA - Home Care Agency	2

Data were collected via telephone and WebEx interviews. Each interview lasted around 35–45 (maximum = 56 min). Six follow-up interviews lasting approximately 9–13 min were completed to verify previously collected information. Three participants agreed to participate in a conference call with the researcher after data analyses to be debriefed on the findings. All interviews were audio-recorded using an encrypted password-protected digital audio recorder to protect participants’ privacy. All audio recordings were professionally transcribed within 24 to 48 h of completion. The transcripts were reviewed for accuracy against the audio recording; when real names were used, they were removed to maintain confidentiality.

Semi-structured interview questions (Table 2) were developed to enable participants to describe the factors they perceived contributed to delayed completion of the RN–BSN program. Several probing questions were used to thoroughly explore the factors that participants reported as significant to their education.

**Table 2:** Semi-structured interview questions

1.	Tell me what made you decide to return to school for RN to BSN education.
2.	Tell me about the most important factors you considered before choosing which school to attend for your RN to BSN education.
3.	Tell me about the experiences that had an impact on the progress you were making while working towards completing your RN to BSN education.
4.	Since you have been in the program, can you tell me what has been happening that caused you to take longer than two years to complete the RN to BSN program?
5.	Tell me what could have been done to help you complete the program on time?
6.	Tell me anything else you would like to share about your experience in the RN to BSN program.

### Data analysis

Thematic content analyses for generating category systems [15] was conducted. This methodology was adopted from literature on content analysis and other sources of qualitative analysis methodology to produce a systematic methodology for recording themes from semi-structured interviews that utilize open-ended questions [32].

The first stage of data analysis began soon after the interviews were completed. The researcher listened to the audio recordings, made notes, and added observations made during the interviews. Then, transcripts were reread to verify the accuracy of the audio recordings, and codes were assigned to the segments, per content analysis [15, 32]. The above iterative process continued until no new codes could be formed from subsequent interviews; then, data collection stopped. Related codes were broadly categorized to form the emergent themes, and participants’ direct quotes are provided.

Creswell [22] suggested that qualitative researchers use at least two of the following eight key strategies developed from Lincoln and Guba’s five criteria for ensuring rigor in qualitative research: prolonged engagement with participants, triangulation, peer debriefing, negative case analysis, reflexivity (bracketing), member-checking, rich description, and external audits. As the data collection and analysis progressed, the author met regularly with a qualitative research expert from another discipline to discuss the developed code lists and to compare the emerging themes with the information from the transcripts. The expert offered insights and alternative views of the codes—challenging the researcher’s interpretations and compelling the author to further evaluate, revise, and consolidate some of the codes and categories. Ongoing discussion with an impartial peer helps researchers “clarify their interpretations of the data, and identify possible sources of bias” ([27], p. 642). After data analysis completion, the researcher discussed the final themes in a 30-min conference call with three participants to ensure credibility [27, 28, 30]. The participant agreed that the findings as presented by the author was an accurate representation of their experiences and descriptions.

## Results

The key findings are shown in Table 3.

**Table 3:** Findings

Themes	Reasons for returning to school	Choice of school and Program	Student related factors	Organizational/Institutional factors	Faculty related factors
	“My job required it;”	Online learning is easier	Financial constrains	Availability of support	Faculty feedback
	Job change	Peer recommendation	Competing responsibilities- family and work schedule	Deceptive marketing strategies	Faculty support/flexibility
**Subthemes**	Career change and job stability	cost	Poor study habits, writing skills and technology challenges	Disregard for prior learning.	Faculty communication
		consideration for prior education	Poor time management	Lack of incentive for educational advancement	

The findings of the study are discussed in two parts to answer three important research questions. First, the findings in response to two major questions i) What factors led to the decision to return to school for an online RN–BSN Program? ii) What were the most important factors online RN-BSN students considered before choosing which school to attend?. Second, the rest of the findings are discussed under the three major themes elucidated in Table 3.

### Reasons for returning to school for RN–BSN program

The most common response to the above question was, “*My job required it;” “I wanted to work in a big hospital, [and] having BSN increases my chances of getting in*;” and *I always wanted to become a nurse practitioner someday—so I have to start somewhere*.” Pauline stated,

*“I have been a nurse for over 15 years; the only reason I returned to school was because I love where I work. I did not want to have to find another job. I was given five years to get the BSN; so, I am using the whole five years.”*

Hannah, who has been a nurse for 21 years stated,

*“I was happy with my job until my hospital decided to go Magnet … I had no intention of going back to school. I tried to find a new job; but no one will pay me what I was making at my current job. So, I went back to school.”*

Some participants went back to school for personal reasons. For Izzy it was to earn the respect of her peers: *“I have been a good nurse for years; then, all of a sudden, I wasn’t good enough because I don’t have a piece of paper with BSN on it … I wasn’t mentally prepared for it; but I am doing it.”*

Another reason was for financial stability and job security. Fernanda stated,

*“I went to nursing school because I needed a career change and stability. I was laid off from my financial job a few years ago. I knew that a BSN was important for that security; but I am in no hurry to get there.”*

The least frequent reason given for returning to school was plans to go to graduate school (*n* = 2). Zumar stated, “I have been going to school for as long as I can remember … I was in no hurry … I plan to go to grad school after I complete the BSN.”

### Choice of school and program

When participants were asked about the most important factors considered when making school and program choices, their responses varied. Some thought completing the RN–BSN program *online would be easy*. Many did not account for the discipline and level of time management required to be successful in an online environment. Izzy stated, *“I really thought that going online will be really easy—less work than face-to-face since I don’t have to take the time to go to class. Boy, was I wrong! It is way too much work.”* Chidi chose a program because she heard from her colleagues that online learning is easier, students can work and complete the assignment at their own pace.

Peer recommendation was the second most common reason cited by participants as influencing their program choices. Tamara explained, *“I was convinced by one of my colleagues to attend the same school he was attending … He made it seem easy … manageable. He promised to help me with technical issues.”* Gina explained, “*… My colleagues told me the program had no exams or quizzes. I hated those when I was doing my ASN [Associate of Science in Nursing].”*

Two of the most significant consideration for schools and programs were cost and consideration for prior education. For some participants, being offered a flat tuition for all the RN–BSN core courses was important; for others, paying the same tuition from the time of enrollment till graduation was important. Danny, who completed an online program in four years, said, *“I chose my program because of the cost. You pay one flat amount of money for all your nursing classes. For $10,000, I can complete 32 credits.”* Fuschia chose a program that offered a tuition lock from enrollment till completion if completed within six years. She stated, *“It was perfect because I needed to take my time and not worry about tuition or fee increases.”*

Brandy’s choice was based on the school that had the most generous transfer policy based on her previous learning*.* She applied to three programs before making a final decision: *“My program took most of my previous credits. I had over 130 credits before I finished my ASN program. The program with the most generous credit transfer was the most logical choice for me*.”

Within the context of the interviews, participants described several factors that contributed to delayed completion or withdrawal from online RN–BSN programs: student-, organizational/institutional-, and faculty-related factors.

### Student-related factors

Student-related factors included participants’ ability to navigate many responsibilities outside of work and school. These included financial constraints, competing responsibilities such as family and work schedule, poor study habits/writing skills and technology challenges, and poor time management.

#### Financial constraints

Almost all participants agreed that BSN education is important, and many reported that getting the degree will offer more job security. However, they also reported financial limitations as a barrier. Six participants who held previous bachelor’s degree did not qualify for federal financial aid. Three participants stated that their current income disqualified them from regular financial aid. Many participants stated that they did not want to incur more students’ loans for nursing education. Two participants stated that they had children in college and were more concerned with their children’s education than their own. For example, Yara stated,

*“I returned to school at the age of later in life to get an associate degree in nursing. That was my end goal … Between my partner and I, we owe over $130,000.00 in student loan [s]. We make a little over that a year. Why would I want to take on more debt if I can complete the program in seven years with little out-of-pocket expense and the tuition reimbursement offered by my employer?”*

Although many participants received some financial assistance from their employers for educational advancement, they felt that it was inadequate to pursue the degree full-time. Some programs also expect the students to pay their tuition within a time frame after course registration. Andy stated,

*“Once you register for the course you have a few weeks to pay … If you don’t pay, they begin to charge you late fees … Sometimes it can take up to six weeks to get the approval for reimbursement after you successfully complete the course. I have other responsibilities like rent and food.”*

The same sentiment was echoed by Ayesha, *“My job offers $2000.00 for tuition after the fact. It will be nice if the money is given at the beginning of the semester, instead of weeks after the course is completed.*”

Scholarships were available to help alleviate the financial burden. However, many participants were not aware of the scholarships or did not apply for various reasons. Yara laughed and stated, *“There isn’t much scholarship out there … the few that I looked at when I first started the program weren’t worth the time or energy.”*

Ayesha compared the scholarship application to filing a tax return:

*“A few scholarships, I thought were worth applying for; but the requirement was like completing a tax return … If I picked [up] 24 extra hours a week at my job, I will make that money in less than four weeks and I will not have to face the shame and disappointment that comes when I get rejected.”*

#### Competing responsibilities: family and work schedule

Many participants had several competing personal responsibilities. All but three participants have children. Some had two or more jobs. Students’ motivation to complete the program was also affected by the value they placed on their educational achievement over other aspects of their lives. For most participants, when family and work responsibilities collided with schoolwork responsibilities, the schoolwork was relegated to the bottom of their priority list.

Family support is an essential factor of students’ academic success [31]. However, some students were the major sources of support for *their* families. Eze stated,

*“I really want to someday get graduate nursing education; but I have to financially support most of my family here and in my country. If they have any problem, I have to drop my course or withdraw from the program to take care of it. Their wellbeing is my priority.”*

Helga reported having two jobs to manage family and school financial responsibilities. *“I have no choice but to work two jobs … I will complete the BSN at some point; but keeping my job and family is more important right now.”* Frances discussed how pregnancy got in the way of completing the program, *“I got pregnant and figured I will take the time off and return after the baby. Before I knew it, two years has passed.”* Unger reported quitting school to keep her family together,

*“Advancing nursing education is not a priority within my family structure. I don’t really have the support of [my] family with nursing school or choosing to return for the BSN … That’s what made me determined to complete the process; but I had to be realistic. I did not want to rock the family boat too much. So, I take it very slow.”*

#### Poor study habits, writing skills and technology challenges

Another factor was students’ approach to learning and the strategies they used to accomplish the tasks related to their education. Students whose motivation for returning to school are extrinsic are more likely to delay completion than those whose intension are more intrinsic [23]. The students in the current study who reported returning to school because their jobs required it dropped registered courses as soon as they faced difficulties. Some reported not fully understanding the time commitment and the discipline requirement for online learning before they signed up. Chidi stated, “*I thought that going online would be easier; but it requires a lot of discipline and self-motivation. I just found all the requirements and self-direction daunting.”* Some participants reported the intensity of written communication and paper writing required in online education versus face-to-face as a barrier. Zumar reported, *“writing papers makes me very anxious and we had a lot of that.”* While Judah explained, *“There is just too much writing in the program. I was not prepared for that. It takes too much time, especially for someone like me who does not enjoy writing.”*

Poor information literacy skills also contributed to the challenges some students faced. Faculty and programs sometimes assume that because students are in RN–BSN programs they are equipped to find evidenced-based information and incorporate them into their work. For some participants, this caused anxiety and undue stress that led to delayed program completion. Lucky stated, *“I just had such [a] difficult time finding the right information for the assignments.”* This was echoed by Alice, “*… It was not until my very last course that I [was] able to connect with the writing center and librarian for help.”*

For some students the RN–BSN program was their first foray into online learning. For these students, navigating the online learning environment was difficult. Although, some programs have learning management platform tutorials and orientations, it was not mandatory. Chidi stated, *“I dropped my first course after the third week because I kept missing assignments. I did not know how to access the course requirements.”* Hannah stated, *“I failed a course because I was not able to use the collaboration function on the course to do my group project. I was ashamed to ask for help and frustrated.”*

#### Poor time management

Poor time management was a significant factor that challenged participants, especially those enrolled in programs with shorter semester time frames of seven to 8 weeks. Andy commented, *“I felt like I was always playing catch up with the assignments. It was very stressful; so, I couldn’t do it full-time.”* From some participants, it was clear that they underestimated the time commitment required to manage online education. Tamara, who chose the program because her friends made it seem easy, stated,

*“I have friends and colleagues who completed the RN–BSN program in record time. They made it seem so easy. I thought it would be the same for me … [but] I lack structure … misjudged the time commitment. I have three young children … I just did not manage my time well.”*

### Organizational- and institutional-related factors

Organizational- and institutional-related factors include availability of support (financial and staff), deceptive marketing strategies, credit transfer issues/disregard for prior learning, lack of incentive for educational advancement, and curriculum factors.

#### Availability of support

Although many programs have academic support in place for students, some students did not know how to access them or that the support was tailored more for traditional students than for online students. Ivan stated that having mandatory online meetings defeated the whole purpose of online learning.

*“I signed up for online education so that I can complete the program on my own schedule; yet, the program requires that we meet at a set time once or twice a week.”*

Lack of institutional financial support was raised by the participants, including those whose employers offered tuition reimbursement. Financial support included limited or no scholarships for RN–BSN students or cumbersome scholarship application requirements. Crystal, who withdrew from her program, stated,

*“The scholarships for RN to BSN were very few; there [was] just too much competition. I was not going to spend time applying to something that I have very little chance of getting. I would rather spend that time writing my paper or picking up extra shift to pay for it myself.”*

Some participants described the requirements for the application of available scholarship as “*not worth the time.”* Others reported that getting assistance with the scholarship application would be helpful. Andy reported, *“I was overwhelmed with the requirements of some of the scholarships, that I did not even consider applying … It will be nice to have help.”* This was echoed by Lucia, *“I wished there was someone to help with scholarship applications. The college website had a list of scholarships … but they did not offer any assistance with that.”*

Lack of academic advising and coaching was also something that frustrated participants. One participant called it “*generic advising*,” meaning that the advisors lacked knowledge about the program and the curriculum, including institutional resources. Alice stated,

*“It will be nice if you have someone to help you navigate all those classes and program requirements after they take your money … If you needed assistance with something, it took days to get meaningful response from anyone.”*

#### Deceptive marketing strategies

Another institutional factor was the lack of transparency in marketing regarding actual program requirements and cost, as well as credit transfers. Twelve participants felt that some programs were not transparent enough in their marketing. Eze explained, *“What they sell you is not really what you get when you start the program”* This sentiment was echoed by Lucia, *“The person I spoke with in admissions told me they will accept all my credits … that was not true … I felt deceived.”*

#### Disregard for prior learning

Many participants were not aware of all the requirements prior to enrolling in the programs. Diana stated,

*“I thought that because we (the ASN program) had [an] articulation agreement with the school, all my credits [would] be accepted; but they were not. I still have to complete 15 more credits plus the BSN courses … I did not know this until I started the program. That meant more money and time.”*

A few participants also reported that their employers pushed some programs over others because of some type of agreement; however, these did not consider their prior learning. Eze wished for *“more options for testing out of some of the courses.”* Programs did not offer more efficient methods of assessing prior learning where available. Hannah explained, “*By the time I complete the portfolio required for the evaluation of prior learning, I would have completed two courses … it just was too much.”*

Another important factor the participants discussed was related to the program curriculum. These included prerequisite courses and stringent program progression plans. What Ivan described as *“too many unnecessary prerequisite courses and strict progression plans,”* caused delays and frustrations. Some felt like they could not take a semester break and be able to pick up from where they left off.

#### Lack of incentive for educational advancement

Although almost all participants agreed that BSN education is important, they also reported that there is very little financial or career incentive to be in a rush to complete the programs. Alice stated, *“I make a decent amount of money without the BSN; so why should I be in a hurry to get that. Moreover, I can run circles around a lot of the nurses who graduate with BSN.”*

Some others talked about their employers not offering any financial incentive for getting the BSN. Fuschia said,

*“… You get very little tuition reimbursement, $1,000.00 a year. The people who went back to school had nothing to show for it but the BSN behind their names … I spent over $15,000.00 for [my] BSN diploma, just to get measly $25.00 added to my pay every two weeks.”*

Ayesha described the only incentive to returning to school saying*, “I am doing it so that I can remain at the same hospital and not lose my seniority.”*

### Faculty factors

Several faculty-related factors contributed to delayed program completion or withdrawal. For some students, it was vital that faculty engaged in consistent, positive, and emotional teacher–student interactions. Perceived poor faculty support contributed to program delay and withdrawal. The subthemes included faculty feedback, faculty support/flexibility, and faculty communication.

#### Faculty feedback

When students lacked self-confidence and perceived that faculty members did not care about them it negatively impacted their program progression. *Lucia stated, “When you are struggling to do your best … and the faculty feedback is rude and judgmental, it makes you question your abilities.”* Alice described her experience with faculty feedback during her first course, *“Some faculty feedback was really discouraging. I already feel bad about a lot of things and I did not have the energy to deal with some of the offensive feedback about my work.”* Cherry echoed this sentiment, *“Some of the comments on my paper made me feel like … stupid … Demeaning faculty comments are not useful. You ask a question about something and you don’t hear back … when you do, it is flippant and disrespectful.”*

#### Faculty support/flexibility

Along with feedback issues, some participants also mentioned the lack of flexibility as a barrier. Especially, when family-related factors were accompanied by lack of flexibility from faculty it led to students dropping the course or sometimes withdrawing from the program. Unger stated,

*“I had a course in the summer semester. I was hoping to catch up; but with my children at home it was challenging going to work, finding childcare, and completing my assignments. I tried to do my schoolwork at night. I asked the faculty for [an] extension on a paper; but she refused … Rather than get an F, I dropped the course.”*

This sentiment was echoed by Helga, “*Sometimes faculty are unrelenting and too strict—they fail to recognize that life happens to students outside of school.”*

The above experience was contradicted by five participants, who reported that their programs and the faculty were very flexible and accommodating to students’ needs. Brandy reported,

*“My program did whatever they could to support the students. In fact, the program director called me when I failed to turn in assignments and did not return emails from the faculty. She wanted to know if there was anything, they can do to help me complete the course I was registered for.”*

Eze stated, *“The program faculty are very supportive; but they can’t solve my financial problems or do anything about all the general education classes required by the university.”*

#### Faculty communication

Poor communication was also described by participants, including not responding to students’ questions, lack of or very late response to emails, lack of clear expectations, and diverse policies across courses. Some participants described difficulties in communicating with faculty who *“were virtually absent—often taking over 72 hours to respond to questions or emails without any explanation or apologies for the delay*.” Another source of frustration for some students was receiving poor grades without reasonable feedback concerning the grade. Helga stated,

*“If you have to wait 48 hours or more for the faculty [to respond to an email], it makes it tough to stay on top of your class work, especially if you are already having a difficult time staying on track.”*

According to Danny, his program “had a lot of part-time teachers; so, we were not a priority. Some of the teachers had no patience, and they took too long getting back to you.” Gina stated, *“Some faculty were unreasonable with the amount of work that they expected the students to complete every week. The workload was simply unrealistic.”*

## Discussion

This study elucidated the complex factors that contributed to the delayed completion of online RN–BSN programs from students’ perspectives. The findings validate and extend those of previous studies without delineating students in fully online programs from hybrid and face-to-face programs. Students’ reasons for returning to school for RN-BSN program are similar to previous cited reasons such as pressure from employers and employer hiring practices resulting from the popularity of Magnet recognition programs job security and advancement [6, 11, 39]. Increased professional values and growth, earning respect from peers, and improving professional identity were reported as reasons for returning to school by only a few participants. This was reported in other studies [25, 40, 47]. Unlike [33, 35, 39], none of the participants in the current study reported increased clinical knowledge and judgment, or improved patient outcomes as reasons for returning to school. Future career plans, support, and encouragement from colleagues, and improving one’s professional identity were reported by some participants, which was also shown in an earlier study [40].

At the time of this study, only four participants had withdrawn from their programs. One on them (Cherry) was planning to return, and three were unsure. In making the decision to return to school and choosing a program, cost was the single most important consideration and barrier reported by participants. Other students’ experiences with the programs was another significant consideration for students when deciding where to go. Previous studies indicated that nurses who had dependent children or relatives or who worked full-time or multiple jobs had greater barriers returning to school for BSN education than did their counterparts ([1, 33, 48]);. Encouraged by colleagues to return to school and finding student friendly programs were also reported by Megginson [40]. Clearly, family and work responsibilities hinder students’ ability to complete a BSN program in a timely fashion.

Previously cited barriers included finances and having the time to balance family, work, and school responsibilities [43, 44, 48, 49]. Delayed program completion was inevitable when some factors were combined with others. Financial constraint was a factor identified by many participants as contributing to their delayed completion. Students were not aware of the financial resources beyond those offered by their employers. Many students did not know that they qualified for undergraduate scholarships, while others felt that the scholarship application process was arduous and not worthwhile. Participants highlighted that receiving assistance with the scholarship applications would alleviate some of the time and financial constraints, which had not been revealed in prior studies. Financial constraints were reported in previous studies as barriers to returning to school for RN-BSN programs [43, 44]. Previous studies did not report students’ perceptions or knowledge related to available scholarships for RN-BSN students. Consistent with other studies that examined the barriers to returning to school [29, 37], writing and appropriate citation of sources posed challenges for some participants. Some of the current study participants reported lack of recognition for previous academic work, necessitating taking more classes thank anticipated. This was in line with poor recognition for prior academic work previously reported [14, 38].

Institutional related factors reported by participants included limited financial support from employers in the form of tuition reimbursement, this was also reported in previous studies [1, 48]. Altmann’s [6] finding—that employers mandated nurses to return to school to further their education but did not offer additional support—did not apply in this study. Many nurses reported that they received some financial support from their employers, and no one reported lack of managerial support as a barrier. In fact, the work-schedule barriers reported were owing to participants’ choices to work more hours to meet their financial and familial responsibilities. Lack of incentive to return to school for a BSN was reported in previous studies [4, 40, 48]. Altmann [6] suggested that the prolonged nursing shortage, lack of a salary increase, and role changes served as deterrents for returning to school. The lack of opportunity for career advancement and little to no financial incentives for earning a BSN degree was cited by some participants as reasons for delayed program completion.

Also noted was a lack of transparency concerning program requirements and overall cost. This lack of transparency has not been reported in previous studies. This often resulted in prolonged program completion time and increased cost for the students. However, Robbins and Hoke [47] noted that their participants appreciated transparent curriculum, with clear degree plans that allowed them to make better plans for program completion. Further, the lack of regulation concerning RN–BSN programs requirement may allow deceptive marketing strategies to continue unchecked.

Nurses returning to school for their BSN degree are interested in finding RN–BSN programs that will respect employer and familial demands on their time and recognize the education and experience they have already acquired [34]. However, the current findings indicate that many of the programs did not recognize nurses’ prior education and experiences, which contributed to completion delays. Despite articulation agreements between two- and four-year colleges offering RN–BSN education, many students had to take many courses to complete the program [40]. A lack of academic advisors also exacerbated this issue for the students in the current study.

Unlike prior studies [29, 40, 49], in which several participants reported fear of technology as a barrier, only four participants in the current study reported major technology-related challenges. Most issues were related to familiarity with the online learning platforms and the lack of uniformity across courses. Those who could accessed the resources found them helpful, which was also found previously [49]. It is essential that the institutional resources available to students be communicated clearly and be made available remotely in fully online programs. Previous studies [26, 29, 44, 47] reported that availability and access to support from academic advisors, librarians, writing center personnel, scholarships, academic support coaches, and individualized academic plans were all significantly related to positive academic experiences and successful program completion. Assessment of and mitigation for the essential skills necessary to be successful in online programs at the beginning of the program were important for students’ success [47].

Some participants reported faculty-related factors that contributed to delayed completion. Faculty flexibility was reported as supportive, while lack of flexibility posed challenges, extended delayed response to emails and inquiries from the faculty or advisor let to frustrations and contributed to delayed program completion. Constructive faculty feedback aimed at encouraging professional growth increased students’ confidence and fostered persistence in the program. Lack of support and poor communication were reported as barrier to program completion in prior studies [5, 20, 29, 47]. An interesting finding was that students perceived that some online faculty were not well-versed in online teaching. They felt that some of their faculty treated the online learning environment same in class learning.

### Limitations

This study had some limitations. Participants may have perceived the delayed completion or withdrawal from the program as a failure, which may have influenced how they responded to some questions. The descriptive research design and small sample size also limits the generalizability of the findings. However, the perceptions of these students cannot be disregarded when exploring factors contributing to delayed program completion or developing support programs to encourage the return to school and the completion of program of study.

### Implications for nursing education

The current study focused on the perception of students enrolled in online RN–BSN programs. The results are significant because they provided detailed information exclusively from the perspectives of online RN–BSN students nurses from various institutions across the U.S. Institutions could use this information when addressing the challenges contributing to delayed completion or withdrawal from their RN–BSN programs. The findings also inform those who advocate for additional funding to support online RN–BSN programs.

Effective online teaching is inherently different from face-to-face teaching. Nurse educators need to be skilled in online teaching provision, including integrating online learning resources without overwhelming the students and without being too rigid in the structure. Providing flexibility and unrestricted access to course materials, which allows students to complete their work on time, is required. Institutions have a responsibility to support their educators in the enhancement of quality online education. Institutions should provide online educators with the resources and necessary training required to be successful in online learning environment.

Employers can incentivize their nurses to complete BSN programs by expanding their roles to include participation in unit and institution-wide committees, leadership roles, and other activities that challenge them to practice beyond their current levels. Academic institutions could facilitate on-time program completion by increasing support services for online learners. Assistance with scholarship applications may alleviate students’ financial burdens.

## Conclusion

Students, faculty members, and institutions all have a role to play in facilitating on-time program completion. Program stakeholders need to be cognizant of the factors that affect students’ program completion. Faculty were perceived as both a challenge and as a support system; thus, it is critical to determine this distinction. Mitigating the barriers to on-time completion of RN–BSN programs will foster timely completion and off-set program withdrawals.

Future researchers should investigate the challenges of online RN–BSN programs from faculty and academic nurse leaders’ perspectives. Study should also investigate what nurse education leaders are doing to mitigate student attrition and to facilitate on-time completion. Future studies can explore what incentives employers are offering non-BSN nurses to return to school. To foster nurses’ motivation to obtain their BSN, it is imperative that stakeholders recognize the barriers that prevent nurses from returning to school and delay program completion and to implement feasible strategies to address them. The current findings can be used to develop strategies to facilitate smooth transition and ultimate on-time completion.

## Supplementary Information

**Additional file 1.** IRB approved Demographic data collection form.

## Data Availability

The datasets generated and/or analyzed during the current study are not publicly available. This is a qualitative interview study with extremely large volumes of transcribed data. The author is willing to share specific transcript upon request. However, the data are available from the corresponding author on reasonable request and approval from the university IRB.

## Abbreviations

AACN: American Association of Colleges of Nursing
AD: Associate Degree
ANA: American Nurses Association
ASN: Associate of Science in Nursing
BSN: Bachelor of Science in Nursing
CCNE: Commission on Collegiate Nursing Education
IRB: Institutional Review Board
IOM: Institute of Medicine
NCLEX: National Council Licensure Examination
RN: Registered Nurse
