# Supplementary material for: Students’ perspectives of factors related to delayed completion of online RN-BSN programs
Source: BMC Nurs. 2021 Apr 7;20:53. doi: 10.1186/s12912-021-00574-7 (PMC8025495; doi:10.1186/s12912-021-00574-7)
Supplement: Supplementary file 1 — Additional file 1. IRB approved Demographic data collection form. [file 12912_2021_574_MOESM1_ESM.pdf]

**IRB approved Demographic data collection form**

| | **Questions** | **Responses** |
|---|---|---|
| | **Pseudonym** | |
| 1. | What is your gender? | |
| 2. | What is your age? | |
| 3. | What is your race or ethnicity? | |
| 4. | How many years have you been a nurse? | |
| 5. | In what type of setting do you primarily practice? | |
| 6. | How long have you been in the RN to BSN program? | |
